# Whole-Body Vibration Effects on Flexibility in Artistic Gymnastics—A Systematic Review

**DOI:** 10.3390/medicina58050595

**Published:** 2022-04-26

**Authors:** Dušan Đorđević, Miloš Paunović, Dražen Čular, Tomislav Vlahović, Miljenko Franić, Dubravka Sajković, Tadija Petrović, Goran Sporiš

**Affiliations:** 1 Faculty of Sport and Physical Education, University of Niš, 18000 Niš, Serbia; zuxxx123@gmail.com; 2 Faculty of Kinesiology, University of Split, 21000 Split, Croatia; dcular@kifst.hr; 3 Department of Traumatology, State Hospital “Sestre Milosrdnice”, 10000 Zagreb, Croatia; t_vlahovic@yahoo.com (T.V.); dubravkasajkovic@gmail.com (D.S.); tadijap@gmail.com (T.P.); 4 Department of Traumatology and Orthopedics, Department of Surgery, Department of Rheumatology, Physical and Rehabilitation Medicine, Clinical Hospital Dubrava, 10000 Zagreb, Croatia; mfranic@kbd.hr; 5 Josip Juraj Strossmayer, School of Medicine, University of Osijek, 31000 Osijek, Croatia; 6 School of Medicine, University of Zagreb, 10000 Zagreb, Croatia; 7 University of Applied Sciences, 10000 Zagreb, Croatia; 8 Faculty of Kinesiology, University of Zagreb, 10000 Zagreb, Croatia; goran.sporis@kif.unizg.hr

**Keywords:** vibration platform, range of motion, improvement, artistic gymnastics

## Abstract

It is well documented that whole body-vibration training has effects on muscle strength and flexibility, blood circulation, decreases pain perception and strengthens bone and tendon. Although whole body-vibration has benefits in athletes’ flexibility, we are not sure what its actual effects are in artistic gymnastics (since they already have stunning flexibility). Hence, the aim of this study was to analyse the studies on whole-body vibration in artistic gymnastics and to present the effects on flexibility. The search and analysis were carried out in accordance with PRISMA guidelines. The databases search (PubMed, Scopus, Google Scholar, Cochrane Library, ProQuest, EBSCOhost and Science Direct) yielded 18,057 potential studies. By the given inclusion criteria (studies from 2005 to 2022; full-text published in English; the study included male and female gymnasts as samples, and that participants were tested for evaluation of flexibility by whole-body vibration method), a total of 9 full-text studies were included, with a total of 210 participants, both male and female. As far as the measured flexibility tests conducted, front split, sit and reach and bridge were evaluated, while obtained results were 9.1–39.1%, 2.79–6.7%, 6.43–7.45%, respectively. All studies have conducted same vibration frequency (30 Hz) with same amplitude of displacements (2 mm), except for the one study who did not show the information of implemented amplitude. After analysing the obtained results, it can be concluded that the usage of whole-body vibration platform shows flexibility improvements in artistic gymnasts, both male and female. In addition, a combination of whole-body vibration and traditional static stretching may enhance the flexibility in artistic gymnasts. However, these results should be taken with caution. Since this review did not reveal the optimal vibrational protocol, it is necessary to invest time during the implementation of various vibrational experimental protocols, so future research is required.

## 1. Introduction

The success in training and talent development depends entirely on a clear understanding of specific performance requirements in a wide range of sports, especially in ones that requires high flexibility, such as artistic gymnastics [1]. To achieve perfect posture and position, requirements of sport uses large ranges of motion in various body joints, to fulfil the technical requirements-criteria of the elements and exercises [2]. In order for the gymnast to successfully perform elements on any apparatus, complete control of the body is required [3,4] along with great elegance [5,6]. Hence, the greatest discriminator refers to flexibility, which distinguishes artistic gymnastics from other sports, as most significant and unique aspect [7]. Flexibility is the ability of the body to carry out the broadest range of motion in the joint space and is supported by and dependent on the elasticity of muscles, tendons and ligaments [8], i.e., the absolute range of motion in joints or a series of joints and muscles length that cross the joint to induce required movements in artistic gymnastics [9].

Whole body-vibration is a type of exercise applied as a modern exercise technique, provided by the amplitude and frequency used within proper ranges and conducted on a special platform [10]. The platform oscillate over a range of frequencies (1–60 Hz) and amplitudes of displacements (1–10 mm), varying according to the product [11]. The plate is oscillating based on two different systems, reciprocating vertical displacements on the left and right side of a fulcrum, and the whole plate oscillating uniformly up and down. In addition, the acceleration can reach 15 g (where 1 g is the acceleration due to the Earth’s gravitational field or 9.81 m/s^2^) [11]. As emerging training method, it has neuromuscular effects on various number of outcomes [12]. The scientific interest in whole-body vibration is generated when mechanical vibrations are transmitted, when a subject is in contact with an oscillating-vibratory platform, which leads to a small but rapid changes in muscle length [11]. It has been used as a safe and accessible way to exercise and important reviews have been published about the use of this exercise to manage diseases and to improve physical conditions of athletes as well [13]. Kelderman [14] have also noted the greatest advantage of the platform, which is achieving significant results and improvements compared to the conventional method. The effects are also shorter training time and execution of movement, increased muscle strength and flexibility, intense stimulation of the neurological system, increased blood circulation, decreased pain perception, causes friction of various types of tissue which results in increased healing of the tissues, strengthens bone and tendon. The mechanical vibration produced in a platform is a physical agent with an oscillatory motion about an equilibrium point [15] and can be determined by biomechanical parameters, such as frequency, amplitude, peak-to-peak displacement and peak acceleration. Besides them, some other parameters must also be considered, as type of vibratory platform (synchronous, alternated or triplanar) [16], duration (working time), time of rest between series, periodicity of the sessions, position adopted by the participants [15], as well as the initial level of flexibility.

Numerous studies suggests that the acute exposure to whole-body vibration has been used as an effective method to improve joint’s flexibility in numerous sports [17,18,19], students [20,21], adults [22] and older adults [23,24]. As far as the examined studies similar to artistic gymnastics (i.e., aesthetic sports), there were also noted flexibility improvements in divers [25], female dancers [26], rhythmic gymnastics [27] and synchronized swimmers [28].

Although whole body-vibration has benefits in athletes’ flexibility, we are not sure what are the actual effects in artistic gymnastics, since they already have stunning flexibility. To the author’s knowledge, there are numerous studies that have dealt with whole-body vibration effects on flexibility in artistic gymnastics (men and women) and there are no conducted systematic review on this topic. Hence, the aim of this study was to systematically review the studies conducted whole-body vibration in artistic gymnastics and to present the effects on flexibility. Likewise, authors have hypothesised that the usage of the whole-body vibration intervention will present positive effects on gymnasts’ flexibility.

## 2. Materials and Methods

### 2.1. Literature Identification

The search and analysis were carried out in accordance with PRISMA guidelines [29,30]. As far as literature identification, the studies had to be published between 2005 and 2022 and the relevant literature for this type of research available in the databases PubMed, Scopus, Google Scholar, Cochrane Library, ProQuest, EBSCOhost, and Science Direct.

To identify relevant articles reporting whole-body vibration effects on flexibility in artistic gymnastics, the following keywords were used: (“whole-body vibration“ OR “whole-body vibration training” OR “vibration” OR “vibration therapy” OR “vibration method”) AND (“stretching method” OR “stretching” OR “flexibility” OR “flexibility enhancement” OR “mechanical stress” OR “physical stress”) AND (“artistic gymnastics” OR “gymnast” OR “competitive gymnasts” OR “male gymnasts” OR “female gymnasts”).

To analyse the obtained data, a descriptive method was used and all titles, abstracts, and full-text articles were reviewed for possible inclusion. The search and evaluation, along with the lists of references from previously assessed and original researches were conducted by two authors (D.Đ. and D.Č.), independently. After that, each author cross-examined the identified studies, which were then taken for further analysis or rejected.

### 2.2. Inclusion Criteria

In order to be included in the final analysis, each study had to meet the following criteria: year of publication (from 2005 to 2022), full-text published in English, the experimental study included male and female gymnasts as sample and that participants were tested for evaluation of flexibility by whole-body vibration method.

### 2.3. Risk of Bias Assessment

The risk of bias was assessed using the Physiotherapy Evidence Database i.e. PEDro scale [31] to determine the study quality and the potential risk of bias. Using checklists, two independent authors (D.Ð. and D.Č.) have assessed it. Concordance between reviewers was estimated using k-statistics data to review the full text and assess relativity and risk of bias. In case of discordance, the obtained data was assessed by the third reviewer (M.P.), who also gave the final decision. The k rate of concordance between reviewers’ findings was k = 0.93.

### 2.4. Data Extraction

After conducting a cross-examination (and only if the data were adequate), the necessary information was extracted. Then, Cochrane Consumer and Communication Review Group’s was applied to extract the characteristics, such as first author and year of publication, sample size, age and training age, experimental intervention such as vibrational frequency, amplitude of displacement, intensity, total experimental duration, in-close device information, protocol exercise(s) and study results.

## 3. Results

### 3.1. Quality of the Included Studies

Based on the total number of included studies and based on the points each study scored on the PEDro scale, assessment scores were finally defined. Maher et al. [32] have already considered that optimal awarded points are between 8–11. But, if the study gains between 0–3 points, that study will be classified with “poor” quality, 4–5 points with “fair” quality, 6–8 points with “good” quality, and 9–10 points with “excellent” quality. Of all included studies in this systematic review, 3 studies showed fair quality, 4 studies showed good quality and 2 studies showed excellent quality. All PEDro scale results are presented in Table 1.

### 3.2. Selection and Characteristics of Studies

**Figure 1:**
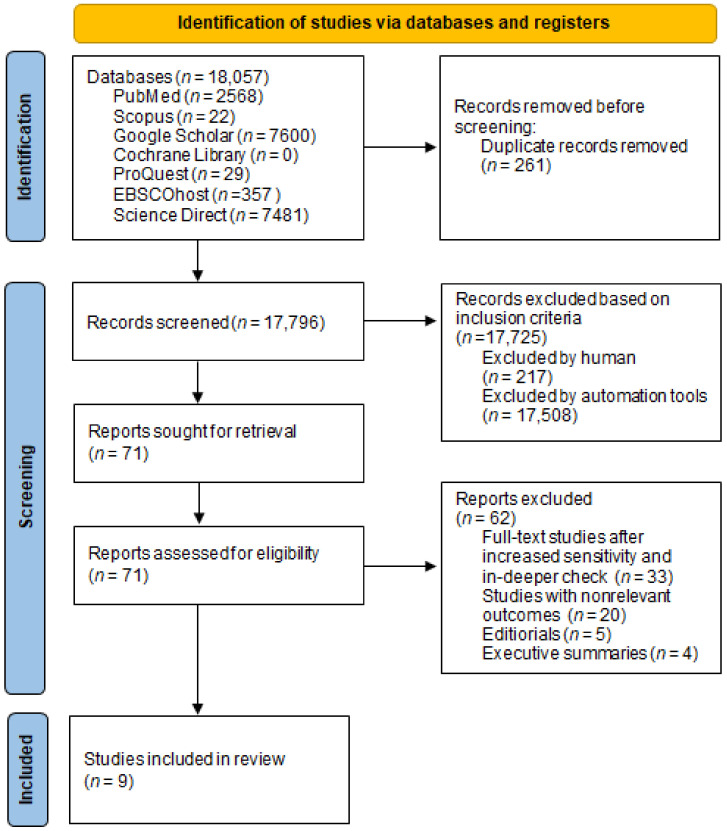
Collecting adequate studies based on pre-defined criteria (PRISMA flow chart).

A search of electronic databases and scanning of reference lists of studies revealed 18,057 studies. Following the review of the duplicates, 261 studies were removed. A total of 17,796 studies were screened, while 17,725 studies were excluded based on the inclusion criteria. Then, 71 full-text studies were taken into consideration and assessed for eligibility, where 62 of them were additionally excluded based on in-deeper check, non-relevant outcomes, editorials and executive summaries. In the end, nine full-text studies were included in the systematic review (Figure 1).

Table 2 show in more detail the studies that met the set conditions and entered the systematic review.

There were a total of 210 participants. The highest number was 52 [36] and the lowest was 10 [33,35], with a total of 48 males and 131 females. Only one study [41] did not showed the gender information. Mixed gender sample were found in three studies [37,40,41]; three studies had male sample of participants [33,35,38], while three studies had a female sample of participants [34,36,39]. The oldest participant was approximately 23 years old [37], while the youngest was 8 years old [36]. The longest practice of gymnastics was approximately 10 years [38,41] while the shortest was approximately 4 years [36,40].

The most measured flexibility test was front split [33,34,35,36,39], sit and reach [38,40,41] and bridge [37]. All studies conducted same vibration frequency (30 Hz) with same amplitude (2 mm), except for the Van Zyl et al. [36] who did not showed the information of implemented amplitude of displacements. Intensity were also various, 2–5 repetitions, 10–45 s, with resting intervals from 5–30 s. Power Plate vibrational devices were used across most of studies [35,36,37,38,40,41], custom made platform was used in one study [33], while in rest of two studies [34,39] the device information was not stated. As far as protocol exercise(s), they were multifarious based on the examined flexibility test. In front split, the vibrated body part was forward posterior lower leg and rear anterior thigh, while in sit and reach, hamstring muscles or examining test with legs on the plate. In bridge, the gymnast placed hands behind the body on vibration device and raised hips from the floor, as removed them towards to the feet to the discomfort point.

Sands et al. [33] have investigated the acute and long-term responses to whole-body vibration and the effects of a four-week intervention, while Dallas et al. [40] did only acute effects, as well as the acute effects of different stretching methods [38,41]. Only Kinser et al. [34] compared the acute effects of a single bout of vibration and stretching on flexibility. Both Van Zyl et al. [36] and Dallas et al. [37] conducted the immediate effects on flexibility. Brooks [39] was the only study that have examined both static and dynamic flexibility.

It should be also mentioned that control groups did not differ from the experimental ones in terms of vibrational protocol, where the device was turned off [33,34,37,39,40] or the control group received no treatment and sat quietly [36].

## 4. Discussion

Based on the review of the relevant literature, this study aimed to systematically review the studies conducted whole-body vibration in artistic gymnastics and to present the effects on gymnasts’ flexibility. The main findings are potential and encouraging benefits of flexibility, in both male and female artistic gymnasts. The findings also suggests that, findings are consistent concerning the whole-body vibration treatment improvement post-intervention compared to the pre-intervention treatment. In addition, this is the first systematic review where the main focus is on artistic gymnasts’ flexibility.

In the field of sports, a lot of technical elements require a high degree of flexibility from athletes in various joints, where studies have shown that the higher the level of flexibility, the better performance level will be shown in sport [42,43]. Hence, artistic gymnastics definitely belongs into the category. The forward split test could be of particular importance for use, since it allows for the simultaneous assessment of flexibility of the adductor and hamstrings muscles, which are among the most often injured muscle groups in the general athletic activities [44,45,46]. Included studies, that have conducted forward split test [33,34,35,36,39], have showed that doing forward split during turned on vibrational platform at different split positions, will allow significant improvements (9.1–39.1%) in the anterior-superior iliac spine. Kinser et al. [34] have reported ≈18.55% improvement, Van Zyl et al. [36] reported 30.38% and both studies implemented combination of stretching with vibrational platform. The participants sample was conducted of girls in both studies and with this regard, same test with boys sample have showed 27.53% improvements [35]. In regard to the obtained results, there should be taking in consideration the gender differences, based on already known fact that women have better flexibility results than men, especially during childhood [47,48]. In addition, all of our identified studies had same vibrational frequency (30 Hz) and amplitude of displacement (2 mm), but the duration, sets and rest periods differed in all of them. That same duration and intensity of flexibility exercises may play an important role, which is why it’s necessary to conduct each exercise in a certain period of time in order to achieve maximum effect on a vibrational platform itself [8]. In addition, it can be assumed that higher amplitudes and frequencies may be more suitable in activating leg muscles, which will result in significant differences [49]. Likewise, some authors [50,51,52] agreed that the higher the amplitude and frequencies are, the greater increases in muscle activity will be, so this factor should be considered in the future.

When the activity is dynamic and demanding, the flexibility is an important aspect of any sports program. According to several authors [53,54,55,56,57], optimum dynamic flexibility provides increased resistance to muscle injury, helping to eliminate awkward or inefficient movement, which leads to improved athletic performance. Only Brooks [39] have realized whole-body vibration protocol with both static and dynamic flexibility and results have showed increased static, but decreased dynamic (split jumps) flexibility. Due to the nature of the split jump (a vertical jump with a split at the height of the jump) the potential deleterious effects of stretching on power [58,59] may have overridden the effects of vibrational platform. According to Page [60], extended static stretching can decrease dynamic flexibility, which further leads to the possible rationale, which is enhanced vibration. The muscles that are contracting during the vertical jump (gluteus maximus, hamstrings, quadriceps and gastrocnemius) are used to produce a powerful upward movement and the facilitation of these muscles to increase power output may inhibit their ability to stretch due to an increased muscle tone [61]. Likewise, there should be also mentioning another disturbing factors, such as overall motor potential and gymnasts weight [62]. Although included studies did not presented the necessary data, further research is needed.

To measure low back and hamstring flexibility, sit and reach test is the most common field test [63], based on its procedure simplicity and facilely to administer [53,64]. In our case, Dallas et al. [38] have showed better improvements in sit and reach using vibrational protocol with static stretching (4.85%, 5.30%, and 5.75%), compared to the vibrational protocol alone (2.81%, 4.15%, 5.79%). In contrary, Dallas et al. [41] have showed only 1.1% improvement. The results are in accordance with Kinser et al. [34], that have been conducted simultaneously stretching and vibration training, as well as previously mentioned ones. But further, the vibration protocol was diverse, so a large number of factors, such as frequency, amplitude or duration of the vibration [65] and rest interval [66] of the intervention protocols may play a significant role in the lasting effects of flexibility that individuals can achieve after different exposures. Also, due to the fact that Dallas et al. [38] and Dallas et al. [41] had older and experienced gymnasts compared to Dallas et al. [40], the results disparity may be also explained by maturation factor which plays crucial role in the flexibility enhancement and it can be main confusing factor [67]. Although some authors [27,68,69,70,71] consider that this test is essential in women’s artistic and rhythmic gymnastics, the justification of improvements could be explained by the motor demands of many elements at the different apparatuses in men’s artistic gymnastics, where the piked position is essential for correct execution [72]. In addition to the above, there should be taken in consideration the fact that any possible stretching related increase in muscle length may not significantly affect the length-tension relationship within the functional limits, particularly in older gymnasts [73].

The flexibility of the spine favours the posture and the amplitude of the movement of the other body segments, being essential in performing the specific elements in artistic gymnastics [1,74]. Dallas et al. [37] have reported 5.79% after 30 min of vibrational protocol in bridge test, while Dallas et al. [38] have showed almost similar results in sit and reach test (6.80%), but after 60 min. The link between the studies is that vibration enhances the stretch reflex loop through the activation of the primary endings of the muscle spindles which influences agonist muscle contraction while antagonists are simultaneously inhibited [17,75]. The benefit of vibration for stretching may be also explained by different mechanisms, such as reduction of phasic and static stretch reflexes, increased pain threshold, increased blood flow with a commensurate increase in temperature and induced relaxation of the stretched muscle [76]. Nevertheless, further investigations are needed.

Although this study provides further support to the vibrational application effects on flexibility on a sample of artistic gymnasts, the coaches should be using this method for overall warming up in all-age competitive gymnasts and combine it with traditional static stretching in order to enhance the stretching methods.

The study limitations are already small number of included studies, as well as the same experimental protocol (30 Hz and 2 mm) in all of them. In addition, only three flexibility tests were conducted, so further studies should be also focusing on a protocol variety, examining the long-term effects, as well as implementing different flexibility tests intended for gymnasts, presented elsewhere [77].

## 5. Conclusions

This is one of the first studies that have summarized the effects of whole-body vibration protocol on artistic gymnasts flexibility. The conclusion drawn from the above mentioned facts would be that the usage of whole-body vibration platform shows flexibility improvements in artistic gymnasts, both male and female. In addition, studies are showing that combination of the whole-body vibration protocol with traditional static stretching may enhance the flexibility in artistic gymnasts, but these results should be taken with caution. Although this review did not reveal an optimal vibrational protocol, the authors believe that it is necessary to invest time during the implementation of various vibrational experimental protocols. In order to make standardized recommend, future research is required.

## Figures and Tables

**Table 1:** PEDro scale results of the included studies.

	Criterion											
Study	1	2	3	4	5	6	7	8	9	10	11	∑
Sands et al. (2006) [33]	Y	Y	Y	Y	Y	N	N	Y	Y	Y	Y	8
Kinser et al. (2008) [34]	Y	Y	Y	Y	Y	N	N	Y	Y	Y	Y	8
Sands et al. (2008) [35]	Y	Y	Y	Y	Y	Y	Y	Y	Y	Y	Y	12
Van Zyl et al. (2011) [36]	Y	Y	Y	Y	N	N	N	Y	Y	Y	Y	7
Dallas et al. (2012) [37]	Y	N	N	Y	N	N	N	Y	Y	Y	Y	5
Dallas et al. (2013) [38]	Y	N	N	N	N	N	N	Y	Y	Y	Y	4
Brooks et al. (2013) [39]	Y	Y	Y	Y	Y	N	Y	Y	Y	Y	Y	9
Dallas et al. (2014) [40]	Y	Y	N	Y	N	N	N	Y	Y	Y	Y	6
Dallas et al. (2014) [41]	Y	N	N	N	N	N	N	Y	Y	Y	Y	4

Legend: 1—eligibility criteria; 2—random allocation; 3—concealed allocation; 4—baseline comparability; 5—blind subject; 6—blind clinician; 7—blind assessor; 8—adequate follow-up; 9—intention-to-treat analysis; 10—between-group analysis; 11—point estimates and variability; Y—criterion is satisfied; N—criterion is not satisfied; ∑—total awarded points.

**Table 2:** Review of included studies.

First Author and Year of Publication	Sample of Participants			Vibration Intervention (VF, AoD, ted, d, PE)	F Tests	Results
	Number and Groups	Age (Years)	Training Age			
Sands et al. (2006) [33]	N-10 (M) E-5 C-5	10.1 ± 1.5	/	A C (NV) E (VF-30 Hz, AoD-2 mm, 4 × 10 s–10 s rest L (4 weeks, 5× a week) ted-4 min d (custom built throught U.S Olympic Committe) PE (forward posterior lower leg, rear anterior thigh)	FS	A Rfs (d = 2.19, p_s_ > 0.84) Lfs (d = 1.67, p_s_ > 0.84) (*p* < 0.05) L * Rfs (d = 1.37, p_s_ ≈ 1.4) (+4.3%) (*p* < 0.05) Lfs (d = 0.84, p_s_ ≈ 0.40) (+4.2%)
Kinser et al. (2008) [34]	N-22 (F) E1-22 E2-7 C2-8	E-11.3 ± 2.6 C-10.6 ± 2.2	E-5.5 ± 2.7 C-5 ± 3.1	C (NV) E (VF-30 Hz, AoD-2 mm 4 × 10 s–5 s rest ted-4 min d (/) PE (forward posterior lower leg, rear anterior thigh)	FS	Rfs * E1 (−18.6 ± 10.4%) (d = 0.67) E2 (−9.1 ± 6.9%) C2 (+2 ± 4.8%) Lfs * E1 (−18.5 ± 7.8%) (d = 0.72) E2 (−10 ± 11.4%) C2 (+1.9 ± 8.2%)
Sands et al. (2008) [35]	N-10 (M)	10.7 ± 0.99	5 ± 1.5	VF-30 Hz AoD-2 mm 2 × 45 s ted ≈ 2 min d (Power Plate Pro 5 Airdaptive) PE (forward posterior lower leg, rear anterior thigh)	FS	* V (*p* = 0.001) Pre (28.8 ± 7 cm) Post (20.8 ± 4.9 cm) Total difference (7.9 ± 3 cm) −27.53% NV Pre (28.4 ± 5.6 cm) Post (24.5 ± 5 cm) Total difference (3.9 ± 1.9 cm) −13.73%
Van Zyl et al. (2011) [36]	N-52 (F) E1-15 E2-9 E3-9 C-19	8–10	≈3	C (NT) E1 (ss-10 min) E2 (VF-30 Hz, 30 s) E3 (ss-10 min followed by wbv- VF-30 Hz, 30 s) d (Power Plate^TM^) PE (distance from the ASIS of the rear leg to the floor with the pelvis)	FS	# £ E1 (*p* < 0.05) Pre (21.3 ± 10.9 cm) Post (17.9 ± 11.4 cm) −15.96% # £ E2 (*p* < 0.05) Pre (16.1 ± 5.6 cm) Post (9.8 ± 6.8 cm) −39.13% # E3 (*p* < 0.05) Pre (15.8 ± 4.1 cm) Post (11 ± 4.2 cm) −30.38% C Pre (20 ± 8.2 cm) Post (18.9 ± 7.6 cm) −5.50%
Dallas et al. (2012) [37]	N-24 E-12 (M-1, F-11) C-12 (F-12)	E-23 ± 2.29 C-20.3 ± 0.78	/	C (NV) E (VF-30 Hz, AoD-2 mm, 4 × 10 s–10 s rest ted-2 min d (Power Plate Pro 5 Airdaptive) PE (from seating position with bent knees, the gymnast placed hands behind the body on vibration device and raised hips from the floor, as removed them towards to the feet to the discomfort point)	B	E Pre (89.59 ± 7.99 cm) * Post 1 min (83.83 ± 8.32 cm) −6.43% Post 30 min (82.92 ± 10.97 cm) −7.45% Post 60 min (83.50 ± 10.37 cm) −6.80% C Pre (93.58 ± 10.35 cm) * Post 1 min (89.42 ± 8.86 cm) −4.45% Post 30 min (90.92. ± 0.21 cm) −2.84% Post 60 min (90.50 ± 13.65 cm) −3.29% * £ Time (F = 24.956, *p* = 0.000) * pre vs. post 1 min (F = 66.573, *p* = 0.000) post 1 vs. post 30 min (F = 0.143, *p* = 0.709) post 30 vs. post 60 min (F = 0.0.09, *p* = 0.926
Dallas et al. (2013) [38]	N-12 (M)	21.88 ± 1.05	8–10	VF-30 Hz AoD-2 mm 5 × 15 s (10 s rest) ted-75 s d (Power Plate^TM^) PE (1–2. From upright position flexed the knees to a squat position, to contact knee extensors, 3. From supine position put and push the hamstring on the patform, 4–5. On the toes to contract calf muscles)	SaR	Day 1 (wbv) Pre (38.83 ± 3.54 cm) * Post 1 min (39.92 ± 3.23 cm) +2.81% * Post 15 min (40.58 ± 2.64 cm) +4.51% * Post 30 (41.08 ± 2.39 cm) +5.79% Day 2 (wbv+ss) Pre (37.75 ± 3.84 cm) * Post 1 min (39.58 ± 4.01 cm) +4.85% * Post 15 min (39.75 ± 3.69 cm) +5.30% * Post 30 (39.92 ± 3.50 cm) +5.75% Condition × trials (F = 1.351, *p* = 0.319) Condition main effect (F = 2.482, *p* = 0.143) * Trial main effect (F = 11.074, *p* = 0.002) * pre vs. post 1 min (F = 38.883, *p* = 0.000)
Brooks et al. (2013) [39]	N-27 (F)	12 ± 2	7.2 ± 2.8	C (NV) E (VF-30 Hz, AoD-2 mm, 4 × 30 s–5 s rest ted-4 min d (/) PE (forward posterior lower leg, rear anterior and posterior thigh)	Sf (FS), Df (Sj)	Sf E Pre (14.9 ± 6.2 cm) * Post (13.3 ± 3.9 cm) (*p* < 0.05) −10.74% C Pre (15 ± 8.8 cm) * Post (13.5 ± 4.7 cm) (*p* < 0.05) −14.56% Df E Pre (166.6 ± 13.5°) * # Post (160.8 ± 18.6°) −3.48% C Pre (167.6 ± 14.7°) * Post (165.3 ± 16.4°) −1.37%
Dallas et al. (2014) [40]	N-34 (M-15, F-19) E-15 C-17	9.22 ± 1.34	3–5	C (NV) E (VF-30 Hz AoD-2 mm 1–2 × 30 s–30 s rest ted ≈ 2 min d (Power Plate^TM^ Next Generation) PE (sit on the floor with legs out straight ahaed, without bending knees, participants is leaning forward without bending their knees and holding the greatest stretch)	SaR	E Pre (29.46 ± 5.96 cm) * Post 1 min (31.33 ± 5.27 cm) +6.35% (F = 20.620, *p* = 0.000) * Post 15 min (31.33 ± 5.38 cm) +6.35% C Pre (29.41 ± 6.56 cm) Post 1 min (30.23 ± 6.67 cm) +2.79% Post 15 min (30.23 ± 6.91 cm) +2.79% Group × time (F = 1.787, *p* = 0.185) * Time (F = 11.885, *p* = 0.000)
Dallas et al. (2014) [41]	N-19	21.83 ± 1.76	8–10	VF-30 Hz AoD-2 mm 3 × 15 s (15 s rest) d (Power Plate^TM^ Next Generation) PE (barfoot sit on the floor with legs out straight ahaed, without bending knees, participants is leaning forward without bending their knees and holding the greatest stretch)	SaR	Day 1 (S + V) £ Pre (35.38 ± 6.08 cm) * £ Post (36.77 ± 6.26 cm) 1.1% * £ Post 15 min (36.77 ± 6.57 cm) 1.1% Day 2 (ss) Pre (36.88 ± 4.68 cm) * Post (39 ± 4.85 cm) 5.7% * Post 15 min (38.83 ± 4.07 cm) 5.3% Day 3 (PNF) # £ Pre (36.94 ± 4.91 cm) * # £ Post (39.44 ± 4.55 cm) 6.7% * # £ Post 15 min (39 ± 4.6 cm) 5.58% * Condition × time (F = 170.77, *p* = 0.034) * Main overall effect in all three conditions (F = 5.52, *p* = 0.0015)

Legend: N—total number of participants, E—experimental group, C—control group, M—male, F—female, F—flexibility, VF—vibration frequency, FS—forward split, ss—static stretching, B—bridge, AoD—amplitude of displacements, A—acute, L—longterm, SaR—sit and reach, Rfs—right forward split, Lfs—left forward split, ASIS—anterior—superior iliac spine, V—vibrated, NV—nonvibrated, NT—no treatment, d—device, PE—protocol exercise(s), wbv—whole-body vibration, wbv+ss—whole-body vibration+static stretching, S+V—stretching on vibration platform, PNF—proprioceptive neuromuscular facilitation stretching, ted—total experimental duration, Sf—static flexibility, Df—dynamic flexibility, Sj—split jump, d—effect size, p_s_—statistical power, * pre—post significant difference, £—significant difference between groups, #—significant difference from C.

## Data Availability

Not applicable.
